# Expression of L-type amino acid transporter 1 as a molecular target for prognostic and therapeutic indicators in bladder carcinoma

**DOI:** 10.1038/s41598-020-58136-x

**Published:** 2020-01-28

**Authors:** Maihulan Maimaiti, Shinichi Sakamoto, Yasutaka Yamada, Masahiro Sugiura, Junryo Rii, Nobuyoshi Takeuchi, Yusuke Imamura, Tomomi Furihata, Keisuke Ando, Kosuke Higuchi, Minhui Xu, Tomokazu Sazuka, Kazuyoshi Nakamura, Atsushi Kaneda, Yoshikatsu Kanai, Natasha Kyprianou, Yuzuru Ikehara, Naohiko Anzai, Tomohiko Ichikawa

**Affiliations:** 1 Department of Urology, Chiba University Graduate School of Medicine, Chiba, Japan; 2 Department of Molecular Oncology, Chiba University Graduate School of Medicine, Chiba, Japan; 3 Department of Tumor Pathology, Chiba University Graduate School of Medicine, Chiba, Japan; 4 Department of Pharmacology, Chiba University Graduate School of Medicine, Chiba, Japan; 5 Bio-system Pharmacology, Osaka University Graduate School of Medicine, Osaka, Japan; 6 Department of Urology, University of Kentucky College of Medicine, Lexington, KY USA

**Keywords:** Cancer of unknown primary, Chemotherapy, Bladder cancer, Bladder

## Abstract

L-type amino acid transporter 1 (LAT1) plays a role in transporting essential amino acids including leucine, which regulates the mTOR signaling pathway. Here, we studied the expression profile and functional role of LAT1 in bladder cancer. Furthermore, the pharmacological activity of JPH203, a specific inhibitor of LAT1, was studied in bladder cancer. LAT1 expression in bladder cancer cells was higher than that in normal cells. SiLAT1 and JPH203 suppressed cell proliferative and migratory and invasive abilities in bladder cancer cells. JPH203 inhibited leucine uptake by > 90%. RNA-seq analysis identified insulin-like growth factor-binding protein-5 (IGFBP-5) as a downstream target of JPH203. JPH203 inhibited phosphorylation of MAPK / Erk, AKT, p70S6K and 4EBP-1. Multivariate analysis revealed that high LAT1 expression was found as an independent prognostic factor for overall survival (HR3.46 P = 0.0204). Patients with high LAT1 and IGFBP-5 expression had significantly shorter overall survival periods than those with low expression (P = 0.0005). High LAT1 was related to the high Grade, pathological T stage, LDH, and NLR. Collectively, LAT1 significantly contributed to bladder cancer progression. Targeting LAT1 by JPH203 may represent a novel therapeutic option in bladder cancer treatment.

## Introduction

Bladder cancer (BC) is the ninth most common malignant tumour worldwide, with 430 000 patients newly diagnosed and 165 000 deaths annually^1^. The pathological type of BC is mainly urothelial cancer ( > 90%) and approximately 70% of patients had non-muscle-invasive BC at diagnosis^2^. These patients have a favorable prognosis with transurethral resection and subsequent intravesical injection therapy, whereas the survival rate of patients with locally advanced and metastatic BC is poor^3^. For metastatic BC patients, platinum-based systematic chemotherapy is the classical treatment, while immunotherapy targeting programmed cell death ligand 1 (PD-L1) blocking antibody was recently approved in Japan^4^. However, drug resistance will occur, and the survival benefit of these agents is not adequate. Their limited efficacy is due to side effects and challenges of drug resistance, leading to treatment failure and require additional treatment options^5^. Therefore, more effective and less toxic therapeutic strategies are needed for the treatment of metastatic BC. Additionally, there are presently no useful diagnostic markers for BC. The urine cytology test is a non-invasive examination, but its sensitivity remains low. Cystoscopy is an essential diagnostic tool but is invasive for patients^5^. Thus, a novel therapeutic approach and biomarker candidates for BC remain a major issue.

Mammalian cells need a large supply of nutrients to grow and reproduce, especially in cancer cells that have lost normal reproductive function. Amino acids are indispensable for the proliferation of cancer cells, and transported to the cell by a selective transporter on the plasma membrane^6^. Several lines of evidence have shown that amino acid transporters are upregulated in cancer cells while supporting large-scale growth and reproduction^7–9^. L-type amino acid transporter 1 (LAT1) transports neutral amino acids into cells in a Na^+^-independent manner^10^. LAT1-mediated leucine uptake interactions with cell growth, transcription, and translation through the mammalian Target of Rapamycin (mTOR) signaling pathway^8,9^. Because many reports have shown that LAT1 is highly expressed in cancer cells, LAT1 has become a novel target for cancers^11–13^. Classically, it has been known that BCH [l- and d- amino acids, 2-aminobicyclo(2,2,1)-heptane-2-carboxylic acid] inhibits both LAT1 and LAT2^14^. When considered as a treatment for cancer, the inhibitory effect of BCH to LAT2 is problematic because LAT2 is mainly expressed in normal cells and BCH may interfere with the function of normal cells^15–17^.

Recently, a selective LAT1 inhibitor named JPH203 [(S)-2-amino-3-(4-((5-amino-2-phenylbenzo [d]oxazol-7-yl) methoxy)-3, 5-dichlorophenyl) propanoic acid] has been created^18–20^. JPH203 inhibits the function of the LAT1 in many types of cancer cells, which functions through the mTOR signaling pathway to block their migration/invasion activities and induce apoptosis^13,21^. Additionally, JPH203 shows significant growth inhibition effects against xenografted human colon or biliary tract cancer cells^22,23^, and its efficacy has been reported in a clinical trial of several cancers^24,25^.

Based on the above-described findings, we hypothesized that LAT1 may be a promising prognostic biomarker as well as a molecular target in BC. Here, we retrospectively characterised LAT1 expression, along with its association with clinical factors in BC tissue. Furthermore, we examined the anti-tumour potential of JPH203, a specific inhibitor of LAT1, in BC cells.

## Results

### Evaluation of LAT1 expression in BC tissue and LAT1 knockdown in BC cell lines

**Figure 1:**
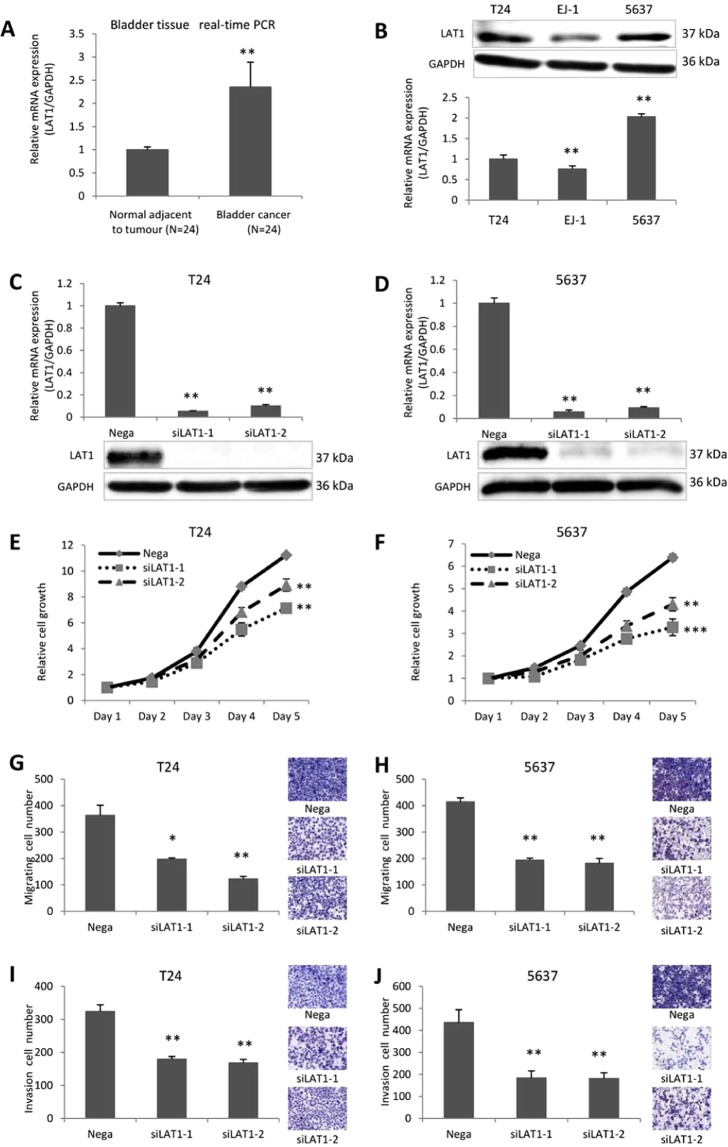
Expression of LAT1 in BC tissue and cells and functional significance of LAT1 in T24 and 5637 cells. LAT1 expression was detected in normal bladder tissue and bladder cancer tissue (**A**). LAT1 protein and mRNA levels were examined in T24, EJ-1, and 5637 cells (**B**). SiLAT1 significantly knocked down LAT1 expression in T24 and 5637 cells (**C** and **D**). SiLAT1 inhibited T24 and 5637 cell proliferation (**E** and **F**), migration (**G** and **H**), and invasion (**I** and **J**). Nega indicates negative siRNA control. Data represent three independent experiments with similar results. P-values were calculated by the Mann–Whitney U-test. *P < 0.05, **P < 0.01, ***P < 0.001.

Significantly higher LAT1 expression was observed in cancer tissues compared with those of adjacent normal tissues (P = 0.0051) (Fig. 1A). Among bladder cancer cell lines, the highest LAT1 protein expression was observed in 5637 cells, followed by T24 cells and EJ-1 cells (Fig. 1B). Based on the basal expression level, T24 and 5637 cells were used for subsequent functional analyses. LAT1 mRNA expression was significantly lower ( < 90%) in siLAT1-transfected than in Negative Control (Nega-transfected) T24 and 5637 cells (Fig. 1C,D) (C: P = 0.0029 and P = 0.0074; D: P = 0.0093 and P = 0.0043; respectively). SiLAT1-transfected cells showed a significant decrease in growth compared with negative control (Fig. 1E,F) (E: P = 0.0070 and P = 0.0080; F: P = 0.0004 and P = 0.0053; respectively). Furthermore, siLAT1-transfected cells also demonstrated significant decreases in migration and invasion activities compared with negative control (Fig. 1G–J).

### Functional role of LAT1 inhibitor JPH203

#### JPH203 suppresses cell growth, migration, and invasion

**Figure 2:**
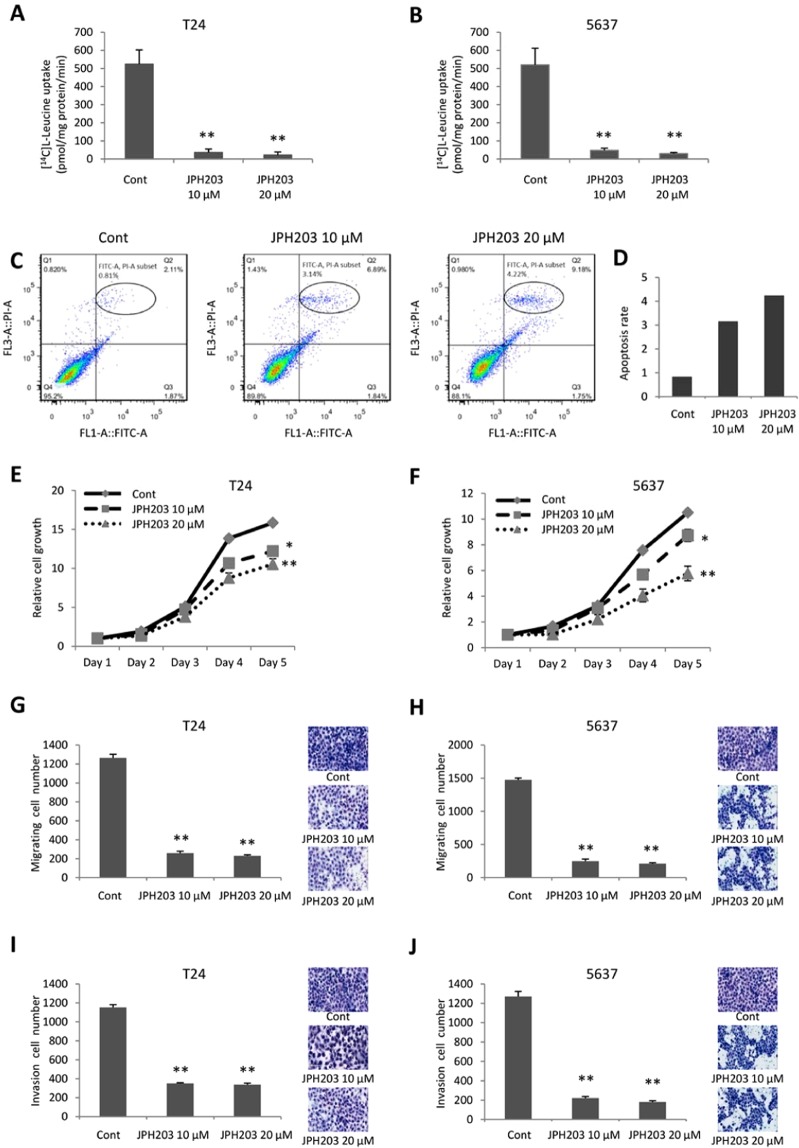
Determination of leucine uptake activities and their sensitivities against JPH203 in bladder cancer cells. Leucine uptake activities in T24 and 5637 cells were determined by transport assays using [^14^C]-leucine (1 µM) in the presence or absence of JPH203 (10 µM and 20 µM). The transport assays were conducted in the presence of Na^+^. JPH203 (10 and 20 µM) inhibited [^14^C] L-leucine uptake (**A** and **B**). Cell apoptosis analysis by Annexin V-FITC/PI staining and flow cytometry. The cell apoptosis rate of T24 cells was slightly increased after JPH203 treatment (10 and 20 µM) (**C** and **D**). JPH203 inhibited T24 and 5637 cell proliferation (**E** and **F**), migration (**G** and **H**), and invasion (**I** and **J**). In all panels, Cont indicates DMSO only. Data represent three independent experiments with similar results. P-values were calculated by the Mann–Whitney U-test. *P < 0.05, **P < 0.01.

Effects of LAT1 selective inhibitor, JPH203, on the viability of T24 and 5637 cells were studied. L-leucine uptake in T24 and 5637 cells was significantly lower than that in DMSO-treated cells ( < 90%) (Fig. 2A,B) (A: P = 0.0025 and P = 0.0038; B: P = 0.0011 and P = 0.0010; respectively). To investigate the mechanistic basis of JPH203-induced inhibition on cell proliferation, we evaluated apoptosis by Annexin V-FITC/PI staining and flow cytometry (Fig. 2C,D). The cell apoptosis rate weakly increased by JPH203 treatment (up to 4.2%) compared with DMSO control (0.8%). JPH203 significantly inhibited the proliferation of T24 and 5637 cells in a concentration-dependent manner (Fig. 2E,F) (E: P = 0.0111 and P = 0.0047; F: P = 0.0180 and P = 0.0079; respectively). Furthermore, JPH203 suppressed the migration and invasion activities of these cells compared with the negative control (Fig. 2G–J).

### Identification of IGFBP-5 by RNA-Seq analysis

**Figure 3:**
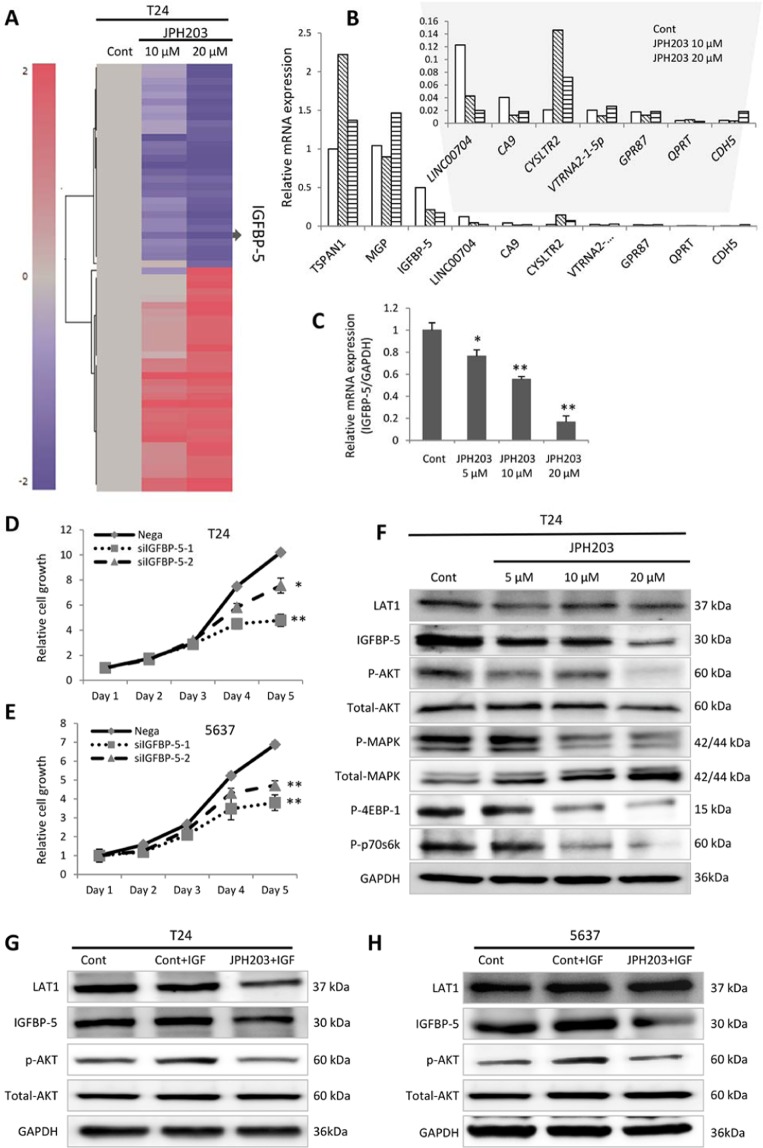
Identification of *IGFBP-5* as a target of JPH203 by RNA-seq analysis. The heat map was generated based on genes changed by JPH203 treatment (10 and 20 µM) (**A**). JPH203 concentration-dependent effect on candidate genes was assessed by real-time PCR (**B**). JPH203 concentration-dependent suppression of *IGFBP-5* was confirmed by real-time PCR (**C**). SiIGFBP-5 inhibited BC cell proliferation (**D** and **E**). After 72 h, JPH203 treatment (5, 10, and 20 µM) inhibited phosphorylation of AKT, MAPK, 4EBP-1 and p70s6k (**F**). The addition of insulin-like growth factor 1 (IGF-1) increased phosphorylated AKT expression, while JPH203 inhibited AKT phosphorylation (**G** and **H**). In all panels, Cont indicates DMSO only, and nega indicates negative siRNA control only. Data represent three independent experiments with similar results. P-values were calculated by the Mann–Whitney U-test. *P < 0.05, **P < 0.01.

To further elucidate the mechanism underlying inhibition by JPH203, we investigated the gene target of JPH203. RNA-seq analysis was performed in two experimental groups cultured with JPH203 (10 and 20 μM) and compared with a control group. Figure 3A shows the heat map generated based on up- and downregulated genes following JPH203 treatment in RNA-seq analysis. The top downregulated target genes were confirmed by real-time PCR analysis (Fig. 3B). Finally, *IGFBP-5* was identified as a target gene of JPH203 with reproducible concentration dependency (5–20 μM) (Fig. 3C).

### Regulation of IGFBP-5 and the mTOR pathway by JPH203

To investigate the proliferative effect of IGFBP-5, the growth of siIGFBP-5-transfected cells was monitored for 5 days. siIGFBP-5-transfected cells showed a significant decrease in growth compared with negative control (Fig. 3D,E) (D: P = 0.0080 and P = 0.0212; E: P = 0.0042 and P = 0.0039; respectively). Because IGFBP-5 modulates IGF-1 signaling, we next investigated potential regulation of IGF-1 signaling, together with mTOR-related signals (Fig. 3F). Western blot analysis indicated marked downregulation of IGFBP-5 and phosphorylated MAPK and AKT, which are related to IGF-1 signals. Additionally, JPH203 treatment downregulated the phosphorylation of ribosomal protein S6K1 and eukaryotic translation initiation factor 4EBP1. Furthermore, JPH203 depletion significantly blocked AKT activation in response to IGF-1 (100 ng/mL) treatment in T24 and 5637 cells (Fig. 3G,H). These results show that JPH203 regulates IGF-1 signals through IGFBP-5.

### Regulation of downstream target gene IGFBP-5 and the mTOR Pathway by LAT1

**Figure 4:**
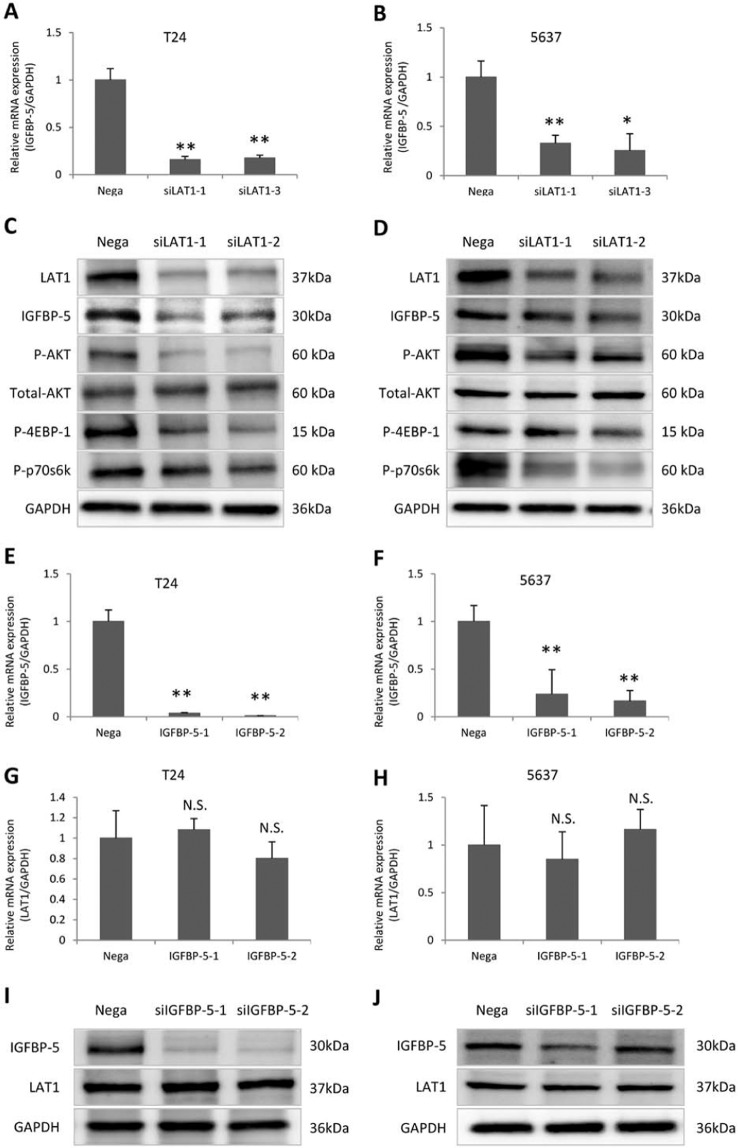
Association between LAT1 and IGFBP-5 expression. The expression of IGFBP-5 in T24 and 5637 cells were inhibited by siLAT1(**A** and **B**). Knocked down the expression of LAT1 inhibited expression of IGFBP-5, phosphorylation of AKT, 4EBP-1 and p70s6k (**C** and **D**). Knocked down the expression of IGFBP-5 in T24 and 5637 cells using siIGFBP-5(**E** and **F**), did not affect the expression of LAT1 in mRNA levels (**G** and **H**) and protein levels (**I** and **J**). Nega indicates negative siRNA control. Data represent three independent experiments with similar results. P-values were calculated by the Mann–Whitney U-test. N.S., no significant difference. *P < 0.05, **P < 0.01, ***P < 0.001.

In order to study the association between LAT1 and IGFBP-5 expression, we studied the effect of siLAT1 on IGFBP-5 expression and the effect of siIGFBP-5 on LAT1 expression. IGFBP-5 mRNA expression was significantly lower in siLAT1-transfected than in Negative Control (Nega-transfected) T24 and 5637 cells (Fig. 4A,B) (A: P = 0.0011 and P = 0.0060; B: P = 0.0082 and P = 0.0210; respectively). Western blot analysis indicated marked downregulation of IGFBP-5, phosphorylated AKT, ribosomal protein S6K1 and eukaryotic translation initiation factor 4EBP1 by siLAT1 (Fig. 4C,D). IGFBP-5 mRNA expression was significantly lower in siIGFBP-5 transfected than in Negative Control (Nega-transfected) T24 and 5637 cells (Fig. 4E,F) (E: P = 0.0049 and P = 0.0049; F: P = 0.0078 and P = 0.0021; respectively). However, siIGFBP-5 did not affect the expression of LAT1 in mRNA levels (Fig. 4G,H) (G: P = 0.7413 and P = 0.1189; H: P = 0.7451 and P = 0.6110; respectively) and in protein levels (Fig. 4I,J).

### LAT1 and IGFBP-5 expression in BC tissue and association with clinical variables

**Figure 5:**
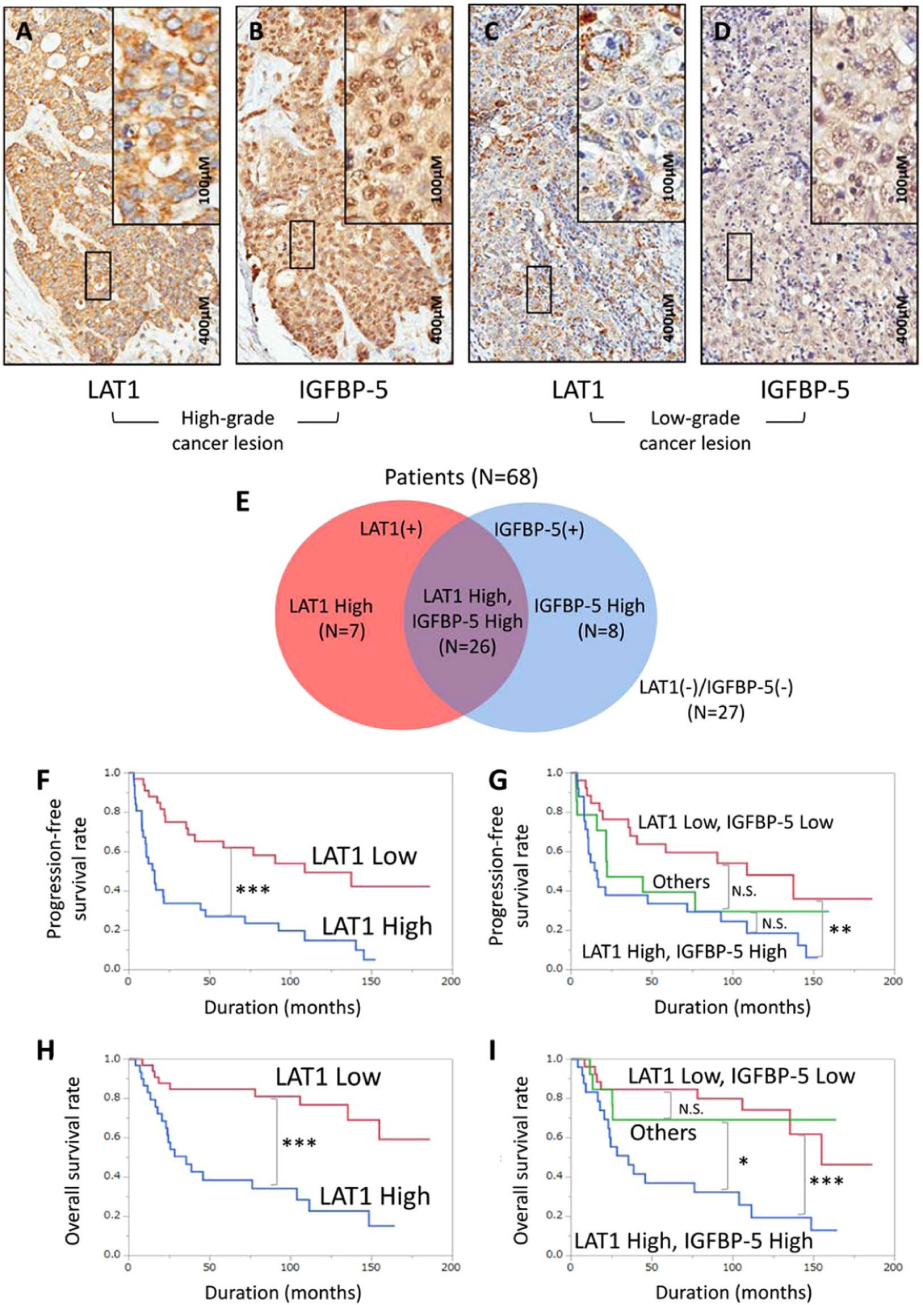
LAT1 and IGFBP-5 immunostaining and score distribution map of BC tissue immunostained for LAT1 and IGFBP-5. Representative images of LAT1 (**A** and **C**) and IGFBP-5 (**B** and **D**) IHC expression. Solid boxes indicate high magnification images of a high-grade cancer lesion (**A** and **B**). Dotted boxes indicate high magnification images of a low-grade cancer lesion (**C** and **D**). Distribution of the number of patients with LAT1 and IGFBP-5 expression (high/low) is indicated inside and outside the circle (**E**). The curve represents the progression-free survival of patients based on LAT1 expression (**F**) and LAT1 and IGFBP-5 expression (**G**). The curve represents the overall survival of patients based on LAT1 expression (**H**) and LAT1 and IGFBP-5 expression (**I**). P-values of the Kaplan–Meier survival analysis were calculated by the log-rank test. N.S., no significant difference. *P < 0.05, **P < 0.01, ***P < 0.001.

To investigate the clinical significance of LAT1, we investigated LAT1 and IGFBP-5 expression in BC specimens by IHC. Positive immunostaining for LAT1 and IGFBP-5 was detected in the cell membrane and cytoplasm. Strong LAT1 and IGFBP-5 immunostaining were detected in cancerous lesions, while noncancerous lesions showed negative or weak immunostaining. We found that, by IHC score, LAT1 and IGFBP-5 were highly expressed in high-grade cancer lesions (26 of 68 specimens, 38.24%). In contrast, low-grade cancers had low LAT1 and IGFBP-5 expression (27 of 68 specimens, 39.71%) (Fig. 5A–D). Overall, 26 of 68 (38.24%) patients had high LAT1 and IGFBP-5 expression, while 27 of 68 (39.71%) patients had low LAT1 and IGFBP-5 expression. Furthermore, 15 of 68 (22.06%) patients had either high LAT1 or IGFBP-5 expression (Fig. 5E).

**Figure 6:**
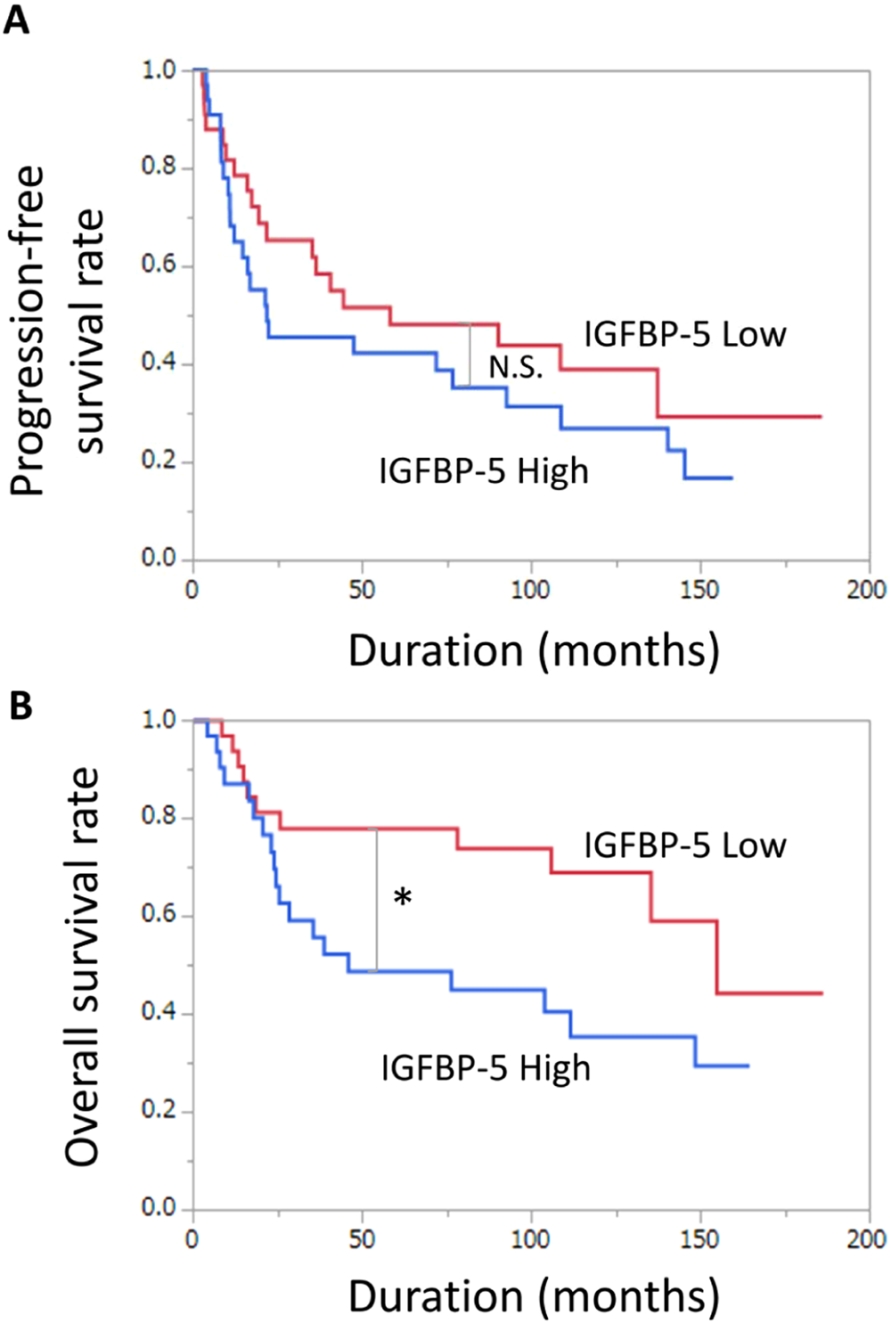
Prognostic significance of IGFBP-5 expression for progression-free survival (PFS) and overall survival (OS). Kaplan–Meier curves demonstrate the prognosis of patients with high and low IGFBP-5 expression for PFS (**A**) and OS (**B**). N.S., no significant difference. *P  <  0.05.

Next, we evaluated the prognostic significance of LAT1 and IGFBP-5 expression in BC patients. Patient characteristics are listed in Table S2. Specimens were divided into two groups based on LAT1 and IGFBP-5 IHC score (median intensity score of 1.14 and 1.62, respectively). The high LAT1 expression group showed a significantly shorter time to progression-free survival (PFS) and overall survival (OS) than the low LAT1 expression group (P = 0.0002 and P < 0.0001, respectively) (Fig. 5F,H). The high IGFBP-5 expression group also showed a significantly shorter OS (P = 0.0422), but not PFS (P = 0.2965) (Fig. 6A,B). When combining LAT1 and IGFBP-5 expression, the high LAT1/high IGFBP-5 expression group showed the worst PFS and OS (P = 0.0075 and P = 0.0005, respectively), followed by the high LAT1/low IGFBP-5 or low LAT1/high IGFBP-5 expression group (others) (P = 0.4212 and P = 0.6262, respectively). In contrast, the low LAT1/low IGFBP-5 expression group showed favourable PFS and OS (Fig. 5G,I).

Next, we investigated associations between clinicopathological BC patient characteristics and LAT1 protein expression. LAT1 expression was a prognostic factor for PFS on univariate analysis (HR 3.14, P = 0.0002). On multivariate analysis, ALP expression was an independent prognostic factor for PFS (HR 2.97, P = 0.0081) (Table 1). LAT1 expression was an independent prognostic factor for OS (HR 3.46, P = 0.0204) (Table 2).

**Table 1:** Predictive factors of PFS.

	Univariate Analysis				Multivariate Analysis		
	Cut off	HR	95% CI	P	HR	95% CI	P
**Age**	69	1.07	0.58–1.96	0.8327			
**Grade**	3	6.03	2.19–24.96	0.0001	3.67	0.57–30.73	0.1719
**pT stage**	3	2.86	1.30–7.54	0.0073	0.81	0.21–3.80	0.7735
**CRP (mg/dL)**	0.60	1.19	0.64–2.26	0.5854			
**LDH (U/L)**	201.00	2.45	1.23–4.96	0.0109	1.90	0.89–4.21	0.0965
**ALT (IU/L)**	9.00	1.01	0.55–1.91	0.9684			
**AST (IU/L)**	17.00	0.94	1.51–1.75	0.8489			
**ALP (IU/L)**	119.00	2.24	1.20–4.31	0.0109	2.97	1.32–6.89	0.0081*
**Hb (g/dL)**	9.90	1.08	0.58–2.04	0.8043			
**Alb (g/dL)**	3.05	1.83	0.88–4.10	0.1093			
**NLR (ng/ml)**	3.53	2.50	1.27–5.20	0.0079	1.16	0.48–3.06	0.7464
**LAT1 score**	1.14	3.14	1.70–5.98	0.0002	1.71	0.66–4.75	0.2702
**IGFBP5 score**	1.62	1.38	0.75–2.55	0.2966			

PFS = progression-free survival, HR = Cox proportional hazard ratio, 95% CI = 95% confidence interval, pT stage = pathological tumour stage, CRP = C-reactive protein, LDH = lactate dehydrogenase, ALT = alanine transaminase, AST = aminotransferase, ALP = alkaline phosphatase, Hb = haemoglobin, Alb = albumin, NLR = neutrophil-to-lymphocyte ratio. *means significant difference.

**Table 2:** Predictive factors of OS.

	Univariate Analysis				Multivariate Analysis		
	Cut off	HR	95% CI	P	HR	95% CI	P
**Age**	69	1.04	0.51–2.17	0.9090			
**Grade**	3	2.47	0.96–8.38	0.0621			
**pT stage**	3	1.29	0.58–3.26	0.5455			
**CRP (mg/dL)**	0.60	0.92	0.43–1.96	0.8168			
**LDH (U/L)**	201.00	2.95	1.27–7.36	0.0114	2.12	0.90–5.42	0.0876
**ALT (IU/L)**	9.00	0.99	0.47–2.14	0.9836			
**AST (IU/L)**	17.00	0.86	0.41–1.82	0.6945			
**ALP (IU/L)**	119.00	2.34	1.11–5.13	0.0248	2.20	0.89–5.80	0.0864
**Hb (g/dL)**	9.90	1.12	0.53–2.37	0.7616			
**Alb (g/dL)**	3.05	1.26	0.53–3.08	0.6046			
**NLR (ng/ml)**	3.53	1.96	0.88–4.65	0.0989			
**LAT1 score**	1.14	4.48	2.08–10.46	< 0.0001	3.46	1.20–11.23	0.0204*
**IGFBP5 score**	1.62	2.23	1.02–4.63	0.0424	1.10	0.42–3.10	0.8526

OS = overall survival, HR = Cox proportional hazard ratio, 95% CI = 95% confidence interval, pT stage = pathological tumour stage, CRP = C-reactive protein, LDH = lactate dehydrogenase, ALT = alanine transaminase, AST = aminotransferase, ALP = alkaline phosphatase, Hb = haemoglobin, Alb = albumin, NLR = neutrophil-to-lymphocyte ratio. *means significant difference.

To understand the clinical characteristics, the relationship between LAT1/IGFBP-5 expression and various clinical factors was determined. High LAT1 expression was associated with advanced pT stage (P = 0.0001), tumour grade (P = 0.0001), high neutrophil-to-lymphocyte ratio (NLR) (P = 0.0007), high LDH (P = 0.0017), and high IGFBP-5 score (P = 0.0001) among BC patients (Table 3). Conversely, high IGFBP-5 expression was associated with the advanced pT stage (P = 0.0003), high LDH (P = 0.0204), high NLR (P = 0.0212), and high LAT1 score (P = 0.0001) (Table S3). When combining LAT1 and IGFBP-5 expression, high LAT1/high IGFBP-5 score correlated with the advanced pT stage (P = 0.0001), advanced tumour grade (P = 0.0026), high LDH (P = 0.0064), and high NLR (P = 0.0070) (Table 4).

**Table 3:** Comparison of clinical factors between LAT1 Low and LAT1 High groups.

	LAT1 Low	LAT1 High	
Age (years)	67.11 ± 8.31	69.70 ± 7.56	0.1850
Grade 3 or greater (%)	57.14	96.67	< 0.0001*
pT stage 3 or greater (%)	47.06	96.97	< 0.0001*
CRP (mg/dL)	1.39 ± 2.55	1.72 ± 2.95	0.6328
LDH (U/L)	177.71 ± 51.99	218.58 ± 36.89	0.0017*
ALT (IU/L)	12.47 ± 7.45	15.03 ± 32.14	0.6529
AST (IU/L)	17.76 ± 8.66	19.23 ± 15.24	0.6325
ALP (IU/L)	137.44 ± 113.93	133.48 ± 53.57	0.8606
Hb (g/dL)	9.74 ± 1.71	10.04 ± 1.70	0.4939
Alb (U/L)	3.65 ± 1.30	3.27 ± 1.29	0.3102
NLR (ng/mL)	2.81 ± 1.46	4.44 ± 1.85	0.0007*
IGFBP-5 staining score	1.02 ± 0.86	2.04 ± 0.85	< 0.0001*

Data are expressed as mean ± standard deviation unless otherwise indicated. LAT1 Low = low intensity of the LAT1 immunoreaction, LAT1 high = high intensity of the LAT1 immunoreaction, pT stage = pathological tumour stage, CRP = C-reactive protein, LDH = lactate dehydrogenase, ALT = alanine transaminase, AST = aminotransferase, ALP = alkaline phosphatase, Hb = haemoglobin, Alb = albumin, NLR = neutrophil-to-lymphocyte ratio. *means significant difference.

**Table 4:** Comparison of clinical factors between other and LAT1/IGFBP-5 high.

	Other	LAT1/IGFBP-5 High	P
Age (years)	67.95 ± 8.13	69.04 ± 7.88	0.5899
Grade 3 or greater (%)	64.29	96.15	0.0026*
pT stage 3 or greater (%)	53.66	100.00	< 0.0001*
CRP (mg/dL)	1.35 ± 2.38	188 ± 3.26	0.4584
LDH (U/L)	183.06 ± 50.23	219.91 ± 39.60	0.0064*
ALT (IU/L)	15.88 ± 27.45	9.41 ± 5.65	0.2796
AST (IU/L)	18.95 ± 14.21	17.50 ± 6.78	0.6523
ALP (IU/L)	134.93 ± 105.49	136.77 ± 47.43	0.9383
Hb (g/dL)	9.75 ± 1.72	10.10 ± 1.69	0.4259
Alb (U/L)	3.57 ± 1.28	3.31 ± 1.35	0.5007
NLR (ng/mL)	3.10 ± 1.49	4.43 ± 2.06	0.0070*
LAT1 staining score	0.66 ± 0.68	2.22 ± 0.40	< 0.0001*
IGFBP-5 staining score	0.98 ± 0.82	2.40 ± 0.50	< 0.0001*

Data are expressed as mean ± standard deviation unless otherwise indicated. Other = low intensity of the LAT1 and IGFBP-5 immunoreaction and only low intensity of the LAT1 or IGFBP-5 immunoreaction, LAT1/IGFBP-5 High = high intensity of the LAT1 and IGFBP-5 immunoreaction, pT stage = pathological tumour stage, CRP = C-reactive protein, LDH = lactate dehydrogenase, ALT = alanine transaminase, AST = aminotransferase, ALP = alkaline phosphatase, Hb = haemoglobin, Alb = albumin, NLR = neutrophil-to-lymphocyte ratio. *means significant difference.

## Discussion

This study proves two novel findings. Firstly, to our knowledge, this is the first study to prove the role of LAT1 in BC. Inhibition of LAT1 suppressed cell proliferation, migration, and invasion *in vitro*. Furthermore, LAT1 expression was associated with OS in BC patients who received total cystectomy. These data indicated a significant contribution of LAT1 in BC progression. Secondly, we identified IGFBP-5 as a novel downstream target of LAT1 inhibition by JPH203. JPH203 inhibited IGF-mediated expression of IGFBP5 and phosphorylation of AKT. IGFBP5 expression was also associated with OS in invasive BC patients.

In previous studies, the selective inhibition of LAT1 by JPH203 has been demonstrated. Several groups reported that JPH203 inhibits the proliferation of cancer cells by inhibiting L-leucine uptake^13,18,26^, LAT1 has been shown to affect cancer cell proliferation through the mTOR signalling pathway in pancreatic cancer^27^, lung cancer^28^, and prostate cancer^6,29^. Leucine is an essential amino acid that must be taken from outside the cells and activates mTOR signals. Increased LAT1 expression in BC could potentially trigger mTOR signaling and further contribute to BC progression and treatment resistance.

The IGF signalling pathway is a complex network that promotes intracellular energy metabolism and protein synthesis. There are two cell surface receptors IGF1R and IGF2R, and two ligands IGF1 and IGF2. The intro is a carrier protein for IGF1. These proteins bind IGF1 with higher affinity and are thus in a key position to regulate IGF signalling. This signalling pathway is essential for normal physiological function, including regulating cell differentiation, proliferation, and apoptosis^30^ Previous data suggested aberrant IGF signalling has been implicated in various cancers, including breast cancer^31,32^ and human colon cancer^33^ and infinite proliferation and division of cancer cells were accomplished by activating IGF1R via the AKT signalling pathway^34^. In addition, IGFBP-5 overexpression indicates a poor prognosis in urothelial carcinoma^35^. In the area of urological cancer, it was reported that IGFBP-5 enhances the anti-apoptotic and mitogenic effects of IGF-I, and accelerates the androgen-independence of PC by the PI3K-AKT protein kinase B signalling pathway^36^. In addition, IGFBP-5 is not expressed in castration sensitive PC cells, but overexpression of IGFBP-5 contributes to androgen-independent PC^36,37^. Our previous report showed that LAT1 expression was low in castration sensitive PC, but it was also highly expressed in androgen-independent PC^29^. These data suggested a similar expression pattern of LAT1 and IGFBP-5 in PC. In this study, both LAT1 and IGFBP-5 were highly expressed in T24 bladder cancer cells. Synchronous expression of two proteins may indicate the active contribution of the LAT1 mediated IGFBP-5 pathway in cancer cells. The mechanism by which the LAT1 blockade targets IGFBP-5 remains to be investigated. Because JPH203 is a LAT1 selective inhibitor. In this report, we are currently considering the presence of the LAT1 binding site within the IGFBP-5 promoter region, which makes it possible for LAT1 to control IGFBP-5 expression. IGF-1 binds to IGFBP in the distal region of the IGFBP-5 promoter. Direct or indirect regulation of IGF-1 through LAT1 blockades, such as cross-talk, maybe another potential underlying mechanism of IGFBP-5 and LAT1 interaction.

A phase I clinical trial of JPH203 in solid cancer was recently completed in Japan. Okano *et al*. presented the results of the phase I study at ASCO 2018 (abstract #2519)^23,38^. The study enrolled 17 patients with colon, biliary tract, pancreas, oesophagus, and breast cancers. Only a moderate grade 3 adverse event was observed with increased levels of alanine aminotransferase (ALT), aspartate aminotransferase (AST), and alkaline phosphatase (ALP) in 12%, 6%, and 12% of patients, respectively. One patient with biliary tract cancer had good disease control for > 25 months, even at the lowest dosage of JPH203 (12 mg/m^2^). The data showed not only a manageable adverse event but also the clinical significance of JPH203 in human patients. We are currently preparing a phase II clinical trial of JPH203 in patients with urological cancer, including BC.

There are several limitations to the present study. Firstly, *in vivo* studies were not carried out. The primary reason is that JPH203 is currently commercially unavailable. We are preparing a material transfer agreement with J-Pharma and will investigate the effect of JPH203 using an *in vivo* BC model. However, as supportive evidence, we have intensively studied the clinical significance of LAT1 in BC patients. Secondly, the number of patients included in this study was rather small. Because this is a pilot study, we are currently conducting a multi-institutional study to verify the prognostic value of LAT1 in BC patients. Thirdly, we have only studied the expression of LAT1 in advanced BC patients who received total cystectomy. The role of LAT1 in the early stage of BC must be investigated. We are presently conducting a prospective study and enrolling patients with early and late-stage BC.

In conclusion, LAT1 was overexpressed in BC, which correlated with poor prognosis. Blockade of LAT1 through JPH 203 inhibited both mTOR and IGFBP-5 related pathways. Current data highlights the potential of LAT1 as a prognostic biomarker and a molecular therapeutic target. To demonstrate clinical benefit, we are currently preparing a phase II clinical trial of JPH203 in patients with BC.

## Materials and Methods

### Ethical approval and consent to participate

The present study was conducted in accordance with ethical standards that promote and ensure respect and integrity for all human subjects and the Declaration of Helsinki. This study was approved by the institutional review board of Chiba University Hospital (approval number 484) and written informed consent was obtained from all patients.

### Reagents and antibodies

Lipofectamine™ RNAiMAX Transfection Reagent, siRNAs siLAT1 (Stealth siRNAs: HSS112004 and HSS188571), siRNAs Insulin-like growth factor-binding protein-5 (siIGFBP-5) (Stealth siRNAs: HSS179821 and HSS105273), and Stealth RNAi™ siRNA Negative Control Med GC Duplex #3 were obtained from Thermo Fisher Scientific (Waltham, MA, USA). Anti-LAT1 (KE023) was obtained from Trans Genic Inc. (Kobe, Japan). Anti-IGFBP-5 (258-270) and insulin-like growth factor 1 (human IGF-1, I3769–50UG) were obtained from Sigma-Aldrich Inc. (St. Louis, MO, USA). Anti-AKT, anti-phosphorylated AKT (Ser473), anti-p44/42 MAPK, anti-phosphorylated p42/44 MAPK, anti-phosphorylated 4EBP-1, and anti-phosphorylated p70s6k were obtained from Cell Signalling Technology Inc. (Danvers, MA, USA). Anti- Glyceraldehyde 3-phosphate dehydrogenase (Anti- GAPDH) was obtained from Ambion Inc. (Carlsbad, CA, USA). LAT1-specific inhibitor, JPH203, was provided by J-Pharma (Yokohama, Japan).

### BC tissue specimens

BC tissue samples were obtained from 68 patients who received radical cystectomy at Chiba University Hospital between 2000 and 2015 and from whom pretreatment information was available. The pathological assessment of these tissues was confirmed. The pathology department of Chiba University Hospital and the tumour pathology department of Chiba University conducted histopathological analyses according to the World Health Organization standard. The clinical and pathological stages were determined by the TNM classification of the International Union Against Cancer. All patients had histologically confirmed BC and tumour samples were examined to ensure that tumour tissue was present in the specimen.

### Cell culture and transfection

T24, EJ-1, and 5637 cell lines derived from human BC were obtained from the Cell Resource Center for Biomedical Research, Institute of Development, Aging and Cancer, Tohoku University (Sendai, Japan). BC cell lines were cultured in RPMI 1640 medium supplemented with 10% foetal bovine serum (FBS) and were maintained in an incubator with a humidified atmosphere of 95% air and 5% CO_2_ at 37 °C. For steroid-free conditions, phenol red-free RPMI 1640 medium was used with charcoal-stripped FBS. BC cells were transfected with siRNA using Lipofectamine™ RNAiMAX reagent (Invitrogen), according to the manufacturer’s instructions.

### mRNA expression evaluation

Total RNA was isolated using the RNeasy® Mini Kit according to the manufacturer’s instructions. Experiments were performed according to a protocol reported previously^39^. First strand cDNA was synthesized with the ImProm-II™ Reverse Transcription System using random primers (Promega, Madison, WI, USA). Real-time reverse transcriptase-PCR was performed with an ABI™ 7300 Real-Time PCR System (Applied Biosystems, Foster, CA, USA). PCR reactions were performed in a final volume of 20 μL of a reaction mixture of SYBR® Green PCR Master Mix (QPS-201, Toyobo, Japan). Relative mRNA expression levels were determined by comparison with GAPDH internal control and plotted as a ratio to GAPDH expression. PCR primers used in this study are listed in Table S1.

### Western blot analysis

Experiments were performed according to a protocol reported previously^29^. Cells were harvested at 72 h after transfection. Protein content was quantified using the bicinchoninic acid protein assay kit (Thermo Fisher Scientific, Waltham, MA, USA), and protein samples (24 mg) were subjected to SDS-PAGE and transferred to Hybond-C membranes (GE Healthcare, Chicago, IL, USA) to manufacturer’s protocol. Following overnight incubation with the respective primary antibody at 4 °C, membranes were exposed to species-specific horseradish peroxidase-labelled secondary antibodies. Signals were detected using the ECL Plus Western Blotting Detection System (GE Healthcare) and visualised using LAS-4000 mini (Fujifilm, Tokyo, Japan).

### Immunohistochemistry (IHC)

I IHC was performed on 4-μm-thick sections of paraffin-embedded specimens using anti-LAT1 and anti-IGFBP-5. Anti-LAT1 antibody (1:200 dilution) and anti-IGFBP-5 antibody (1:200 dilution) at 4 °C in a moist chamber overnight. Then treated with the secondary antibody (biotin-labelled antibody) from the SAB-PO (MULTI) kit (Nichirei, Tokyo, Japan), followed by colour development with 3,3′-iaminobenzidine tetrahydrochloride of the DAB Substrate Kit (Nichirei, Tokyo, Japan). Detailed experimental methods have been previously reported^39^.

To quantify LAT1 and IGFBP-5 protein expression in these components, we accord a previously described IHC scoring^40,41^. The mean percentages of LAT1- and IGFBP-5-positive tumour cells were determined in at least five random fields per section at 400 × magnification. As a negative control, triplicate sections were immunostained without exposure to primary antibodies or secondary antibodies, thus confirming the staining specificity (Fig. S1A). The intensity of the LAT1 and IGFBP-5 immunoreaction was scored to produce respective IHC scores as follows: 0, no staining; 1 + , very weak; 2+, moderate; and 3+, strong staining (Fig. S1B,C). IHC scores were calculated as follows: IHC score = 1 × (mean percentage of weakly stained cells in the field) + 2 × (mean percentage of moderately stained cells in the field) + 3 × (mean percentage of intensely stained cells in the field). Median LAT1 and IGFBP-5 IHC scores were defined as positive (1.14 and 1.62, respectively). Two independent investigators (SS and MM) blinded to patient clinical status scored each specimen.

### Growth assay

All sets of cells were transfected with siRNA using Lipofectamine™ RNAiMAX Transfection Reagent according to the manufacturer’s instructions and then maintained for 5 days. To study the effect of JPH203 treatment, T24 or 5637 cells were seeded onto wells of 96-well plates. After 24 h, the medium was changed to the indicated concentrations of JPH203 for 5 days. Two sets of independent experiments were performed for each indicated time point, at which cells were trypsinized and counted.

### Cell migration and invasion assay

Falcon® Permeable Support for 24-well Plate with 8.0 µm (353097, Corning, Corning, NY, USA) was used for the migration assay, and Matrigel™ invasion chamber 24-well 8 µm (354480, Corning) was used for the invasion assay. The essential parts of the experiments have been described previously^29^. T24 and 5647 cells (5 × 10^4^) were seeded in the upper chamber and incubated for 48 hours with serum-free RPMI1640 medium. Then, stained using the Diff-Quik kit (16920, Funakoshi Co, Tokyo, Japan). The cell numbers in five different fields were calculated under a microscope for each well.

### [^14^C] L-leucine uptake assay

To examine the inhibition of amino acid transport by JPH203 in T24 and 5637 cells, uptake experiments were performed using [^14^C] L-leucine, which is a prototypical LAT1 substrate. Cells were seeded into wells of 24-well plates and incubated at 37 °C for 2 days. After removal of the medium, cells were washed twice with Hanks’ Balanced Salt Solution. Thereafter, cells were incubated with uptake medium containing 1 µmol/L [^14^C] L-leucine at 37 °C for 1 min. [^14^C] L-leucine uptake was terminated by washing cells twice with ice-cold Hanks’ Balanced Salt Solution and solubilisation with 0.1 N NaOH. Cell lysates were mixed using a scintillator and substrate accumulation was measured by counting radioactivity by liquid scintillation counting. The experiment was performed as previously reported^22^.

### Annexin V-FITC apoptosis assay

Apoptosis was evaluated in T24 cells using JPH203 and dimethyl sulfoxide (DMSO) as control for 5 days. The Annexin V-FITC Apoptosis Detection Kit (Abcam, Carlsbad, CA, USA) was used according to the manufacturer’s protocol. Briefly, transfected cells were incubated with Annexin V-FITC and propidium iodide (PI; Wako Pure Chemical, Tokyo, Japan) for 5 min in the dark. Thereafter, apoptotic cells were detected by flow cytometry.

### RNA sequencing

According to previously described RNA sequencing with minor modification^42^. Total RNA was extracted from BC cells using the RNeasy Mini Kit (Qiagen, German), and cDNA was synthesized using the SMART-Seq v4 Ultra Low Input RNA Kit for Sequencing (Clontech, Palo Alto, CA, USA). ds-cDNA was fragmented using the S220 Focused-ultrasonicator (Covaris, Woburn, MA, USA), then cDNA libraries were generated using the NEBNext® Ultra™ DNA Library Prep Kit (New England Biolabs, Beverly, MA, USA). Sequencing was performed using the HiSeq. 1500 system (Illumina, Santiago, CA, USA) with a single-read sequencing length of 60 bp. TopHat (version 2.0.13; with default parameters) was used to map to the reference genome (UCSC/mm10 or UCSC/hg19) with annotation data from iGenomes (Illumina). Then, gene expression levels were quantified using Cuffdiff (Cufflinks version 2.2.1; with default parameters).

### Statistical analysis

Univariate and multivariate Cox proportional models were used for statistical analyses. Survival curves were obtained using the Kaplan–Meier method and differences in survival rates between LAT1-positive and -negative cases, and between IGFBP-5-positive and -negative cases, were compared using the log-rank test. Statistical calculations were performed using JMP® Pro 13, version 13.0.0 (SAS institute, Cary, NC, USA). Significance was considered to exist at P < 0.05.

## Supplementary information

Table S1, Table S2, Table S3, Figure S1.
