# Supplementary material for: Expression of L-type amino acid transporter 1 as a molecular target for prognostic and therapeutic indicators in bladder carcinoma
Source: Sci Rep. 2020 Jan 28;10:1292. doi: 10.1038/s41598-020-58136-x (PMC6987139; doi:10.1038/s41598-020-58136-x)

Title

Expression of L-type amino acid transporter 1 as a molecular target for prognostic and therapeutic indicators in bladder carcinoma

Maihulan Maimaiti[1], †Shinichi Sakamoto[1*], Yasutaka Yamada[1], Masahiro Sugiura[1,2], Junryo Rii[1], Nobuyoshi Takeuchi[1,3], Yusuke Imamura[1], Tomomi Furihata[4], Keisuke Ando[1,4], Kosuke Higuchi[1,4], Minhui Xu[5], Tomokazu Sazuka[1], Kazuyoshi Nakamura[1], Atsushi Kaneda[2], Yoshikatsu Kanai[5], Natasha Kyprianou[6], Yuzuru Ikehara[3], Naohiko Anzai[4], Tomohiko Ichikawa[1]

[1] *1 Department of Urology, Chiba University Hospital, Chiba, Japan*

[2] *Department of Molecular Oncology, Chiba University Graduate School of Medicine, Chiba, Japan*

[3] *Department of Tumor Pathology, Chiba University Graduate School of Medicine, Chiba, Japan*

[4] *Department of Pharmacology, Chiba University Graduate School of Medicine, Chiba, Japan*

[5] *Bio-system Pharmacology, Osaka University Graduate School of Medicine, Osaka, Japan*

[6] *Department of Urology, University of Kentucky College of Medicine, Lexington, KY, USA.*

*Corresponding: Shinichi Sakamoto

Chiba University Graduate School of Medicine

1-8-1 Inohana, Chuo-ku, Chiba-city, Chiba 260-8670, Japan

Tel: +81-43-226-2134 Fax: +81-43-226-2136

Email: rbatbat1@gmail.com

†Equal study contribution

Table S1. Primer sequences for real-time PCR

**Gene**	**Primer Type**	**Sequence (5′ to 3′)**
LAT1	**Forward**	AGGAGCCTTCCTTTCTCCTG
	**Reverse**	CTGCAAACCCTAAGGCAGAG
IGFBP-5	**Forward**	GGAGGAGCCGAGAACACTG
	**Reverse**	GCGAAGCCTCCATGTGTC
TSPAN1	**Forward**	CGTTGTGGTCTTTGCTCTTG
	**Reverse**	TTCTTGATGGCAGGCACTAC
MGP	**Forward**	AGTCCAAGAGAGGATCCGAG
	**Reverse**	ATAAACCATGGCGTAGCGTT
LINC00704	**Forward**	TGCGTTCAGTAAAACGGGCA
	**Reverse**	TGTGGGAAATGCAGGGTTCT
CA9	**Forward**	GCCTTTGAATGGGCGAGTG
	**Reverse**	CCTTCTGTGCTGCCTTCTCATC
CYSLTR2	**Forward**	TCATAAGCACGCTTCCCTTC
	**Reverse**	CACTCCTGATGCTGGTGACA
VTRNA2-1-5p	**Forward**	CGGGTCGGAGTTAGCTCA
	**Reverse**	TGCGAATACCTCGGACCCTG
GPR87	**Forward**	GATAAGCATTGATGCCTATCTGAAGGTG
	**Reverse**	CACCTTCAG ATA G GCATCAATGCTTATC
QPRT	**Forward**	ATGGACTTCTGCTCTCGCTGGTAG
	**Reverse**	TCATTCCTTTGTTGCCACCG
CDH5	**Forward**	CCTACCAGCCCAAAGTGTGT
	**Reverse**	GACTTGGCATCCCATTGTCT
GAPDH	**Forward**	CCACCCATGGCAAATTCC
	**Reverse**	TGATGGGATTTCCATTGATGAC

Table S2. Patient characteristics

**Clinical factors (n=68)**	**Median (range) or n (%)**	**Age (years)/Median (range)**
**Age (years)**	69 (42–83)	
**Grade**		
**2**	16 (23.53%)	68.5 (48–76)
**3**	52 (76.47%)	69 (42–83)
**pT stage**		
**0**	2 (3.00%)	68.5 (68–69)
**1**	4 (5.97%)	59.5 (48–69)
**2**	13 (19.04%)	71 (63–77)
**3**	37 (55.22%)	69 (42–83)
**4**	11 (16.42%)	68 (46–74)
**CRP (mg/dL)**	0.6 (0–15.40)	
**LDH (U/L)**	201 (82–298)	
**ALT (IU/L)**	9 (3–186)	
**AST (IU/L)**	17 (7–94)	
**ALP (IU/L)**	119 (26–646)	
**Hb (g/dL)**	9.90 (6.20–14)	
**Alb (g/dL)**	3.20 (1.30–7.10)	
**NLR (ng/mL)**	3.53 (1.08–9.18)	
**LAT1 score**	1.14 (0–2.80)	
**IGFBP-5 score**	1.62 (0–3)	

pT stage = pathological tumour stage, CRP = C-reactive protein, LDH = lactate dehydrogenase, ALT = alanine transaminase, AST = aminotransferase, ALP = alkaline phosphatase, Hb = haemoglobin, Alb = albumin, NLR = neutrophil-to-lymphocyte ratio

Table S3. Comparison of clinical factors between IGFBP-5 Low and IGFBP-5 High groups

	IGFBP-5 Low	IGFBP-5 High		
**Age (years)**	69.09 ± 7.01	67.65 ± 8.91	0.4613	
**Grade 3 or greater (%)**	67.65	85.29	0.0833	
**pT stage 3 or greater (%)**	52.94	90.91	0.0003	*
**CRP (mg/dL)**	1.52 ± 2.59	1.60 ± 2.93	0.9015	
**LDH (U/L)**	181.39 ± 49.96	212.25 ± 44.83	0.0204	*
**ALT (IU/L)**	17.09 ± 30.90	10.19 ± 7.19	0.2227	
**AST (IU/L)**	19.70 ± 15.73	17.19 ± 6.86	0.4102	
**ALP (IU/L)**	144.36 ± 115.03	126.47 ± 52.68	0.4255	
**Hb (g/dL)**	9.72 ± 1.62	10.06 ± 1.79	0.4231	
**Alb (U/L)**	3.36 ± 1.23	3.58 ± 1.38	0.5699	
**NLR (ng/mL)**	3.03 ± 1.42	4.17 ± 2.05	0.0212	*
**LAT1 staining score**	0.72 ± 0.74	1.80 ± 0.85	<0.0001	*

Data are expressed as mean ± standard deviation unless otherwise indicated. IGFBP-5 Low = Low intensity of the IGFBP-5 immunoreaction, IGFBP-5 high = High intensity of the IGFBP-5 immunoreaction, pT stage = pathological Tumor stage, CRP = C-reactive protein, LDH = lactate dehydrogenase, ALT = alanine transaminase, AST = aminotransferase, ALP = alkaline phosphatase, Hb = haemoglobin, Alb = albumin, NLR = neutrophil-to-lymphocyte ratio

Figure S1. LAT1 and IGFBP-5 IHC-positive and -negative control of BC tissue immunostained for LAT1 and IGFBP-5. A comparative trial with no primary antibody and no secondary antibody was performed (A). The IHC scoring criteria for LAT1 and IGFBP-5 are presented in B and C, respectively.

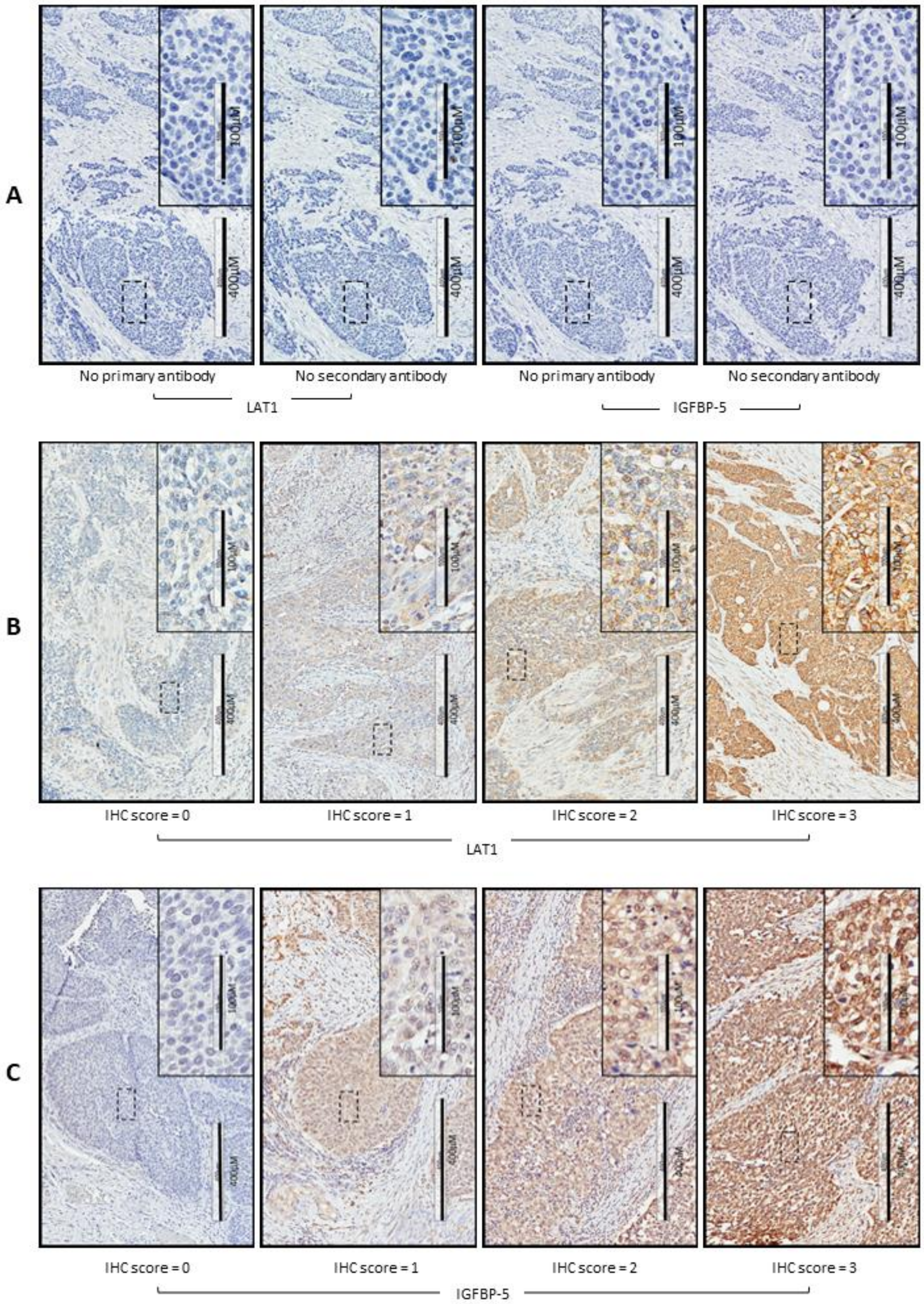

Supplement: Supplementary file 1 — Table S1, Table S2, Table S3, Figure S1. [file 41598_2020_58136_MOESM1_ESM.pdf]
